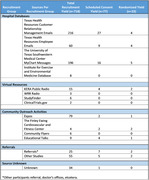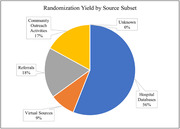# Recruitment Strategies and Preliminary Results from the IPAT Study

**DOI:** 10.1002/alz.087551

**Published:** 2025-01-09

**Authors:** Jordan Zaenglein, Tristyn Hall, Margaret McGregor, Anna Tomlinson, Christopher Reagin, Stephen Mathison, Solymar Rivera‐Torres, Rong Zhang

**Affiliations:** ^1^ Institute for Exercise and Environmental Medicine, Dallas, TX USA; ^2^ Institute for Exercise and Environmental Medicine, Texas Health Presbyterian Hospital, Dallas, TX USA; ^3^ UTSW Medical Center/IEEM, Dallas, TX USA

## Abstract

**Background:**

The Impact of Intensive Treatment of Systolic Blood Pressure on Brain Perfusion, Amyloid and Tau in Older Adults (IPAT) is an ongoing randomized control trial (RCT) for prevention of Alzheimer’s disease (AD) (NCT05331144). The primary outcome of the trial is to assess the impact of intensive lowering of high systolic blood pressure (SBP) on the accumulation of brain amyloid in older adults for a study duration of 2 years. Study recruitment started on October 25^th^ of 2022 and will end in September of 2025. The goal is to enroll 180 participants. Inclusionary criteria include participants who are between the ages of 60 and 85 and have a systolic blood pressure (SPB) of 130mmHg or greater. Exclusionary criteria include those who have a history of major cerebrovascular disease, a diagnosis of Alzheimer’s disease and related dementias (ADRD), other major neurologic diseases, or cannot perform MRI.

**Methods:**

A multimodal recruitment approach was implemented which includes hospital databases, virtual sources, referrals, and community outreach activities (Table 1). The recruitment response yield, scheduled consent visit yield, and randomization yield from October 2022 to December 2023 were analyzed across the recruitment source subgroups.

**Results:**

The total recruitment response was 718 potential participants. Out of 718 potential participants, 77 individuals had an informed consent appointment scheduled (10.7%) and 23 of the 718 individuals were randomized (3.2%). The source subset that produced the highest yield for total recruitment response was hospital databases (67%). The source subset that produced the highest yield for consent visits scheduled was hospital databases (68%). The source subset that produced the highest yield for randomizations was hospital databases (56%) (Figure 1).

**Conclusions:**

The IPAT Study had a success with total recruitment yield from the hospital database sources, but less success with virtual resources, referrals, and community outreach activities. However, a multimodal recruitment approach is essential to meet the overall enrollment goal of the project.